# Urokinase redux: route, compartment, access, and pharmacological evolution from native urokinase to recombinant prourokinase

**DOI:** 10.3389/fcvm.2026.1904249

**Published:** 2026-09-24

**Authors:** Xuxu Xu, Shepeng Wei, Jing Bao

**Affiliations:** 1 Department of Neurosurgery, Yangpu District Shidong Hospital of Shanghai, Yangpu District, China; 2 Department of Neurosurgery, University of Shanghai for Science and Technology, Shanghai, China

**Keywords:** catheter-directed thrombolysis, chronic subdural hematoma, intracerebral hemorrhage, local fibrinolysis, urokinase

## Abstract

Urokinase is often regarded as an older thrombolytic displaced by fibrin-selective agents in reperfusion medicine. Yet its decline has been uneven. This mini-review reframes its contemporary role through three determinants: route, compartment, and access. The systemic role of native urokinase has receded, particularly in stroke. Adjunctive thrombolysis after endovascular reperfusion remains unsettled: POST-UK and POST-TNK were neutral, whereas PEARL and ANGEL-TNK reported higher 90-day excellent-outcome rates. Trial differences preclude a uniform conclusion or generalization to primary intra-arterial thrombolysis and local fibrinolysis. Native urokinase retains a niche when delivered directly into clot-containing compartments or established catheters, including intracranial blood collections, chronic subdural hematoma, selected venous thromboembolism, loculated pleural disease, and catheter-related thrombotic dysfunction. In these settings, it functions as a low-cost, familiar, locally delivered fibrinolytic that facilitates clot clearance or drainage. Supporting evidence remains heterogeneous and often derives from retrospective cohorts, small studies, and variable protocols. Recombinant human prourokinase should be distinguished pharmacologically and clinically from native urokinase. As a zymogen precursor preferentially activated at the fibrin-rich thrombus surface, it represents a renewed systemic reperfusion pathway. Following the historical proof of concept provided by PROACT II, PROST and PROST-2 showed intravenous recombinant human prourokinase to be non-inferior to alteplase for excellent 90-day functional outcomes; PROST-2 additionally reported lower rates of symptomatic intracranial hemorrhage and major bleeding. External generalizability and direct comparison with tenecteplase remain unresolved. The contemporary urokinase pathway therefore reflects a dual evolution: native urokinase retains focused local and access-driven roles, whereas recombinant prourokinase has re-entered systemic reperfusion therapy.

## 1 Introduction

Urokinase is often regarded as an older thrombolytic displaced by more fibrin-selective agents. Yet its contemporary trajectory is not uniform. Native urokinase remains inexpensive, familiar, and widely available in China and other resource-conscious settings, where its clinical value depends on route, compartment, and access.

Its routine systemic role has narrowed. Intravenous native urokinase may remain a pragmatic fallback when alteplase or tenecteplase is inaccessible (1). For post-thrombectomy adjunctive thrombolysis, the randomized evidence is heterogeneous: POST-UK and POST-TNK were neutral after near-complete or complete reperfusion, whereas PEARL and ANGEL-TNK reported higher 90-day rates of excellent outcome in broader eTICI 2b-3 populations (2–5). Differences in reperfusion thresholds, thrombolytic agents and doses, and cointerventions preclude a uniform conclusion. These findings address specific post-reperfusion settings and should not be generalized to the broader urokinase-prourokinase pathway.

Native urokinase may be more rational when delivered directly into a clot-containing compartment or catheterized thrombus. Relevant applications include subdural fibrinolysis (6, 7), catheter-directed thrombolysis for selected deep vein thrombosis (8), and intrapleural fibrinolysis (9). Related stroke evidence includes primary intra-arterial recombinant prourokinase (10) and post-reperfusion adjunctive thrombolysis with urokinase, tenecteplase, or alteplase (2–5), while separate neurosurgical literature addresses intraventricular and intracavitary native urokinase delivery (11–14).

This mini-review therefore distinguishes native urokinase from recombinant prourokinase and examines how agent, route, compartment, treatment stage, and access define their contemporary clinical roles.

## 2 Stroke reperfusion: agent and treatment stage

Acute ischemic stroke illustrates why native urokinase and recombinant prourokinase require separate evaluation. Intravenous native urokinase remains a low-cost fallback in China and other resource-conscious settings. A large retrospective comparison found broadly similar functional outcomes and intracranial hemorrhage rates with native urokinase and alteplase, but more extracranial bleeding with urokinase, particularly at higher doses (1). Native urokinase may therefore be pragmatic when alteplase or tenecteplase is inaccessible, but is not the preferred systemic agent when fibrin-selective alternatives are available.

PROACT II provides important historical evidence for primary intra-arterial recombinant prourokinase. In 180 patients with angiographically confirmed middle cerebral artery occlusion treated within 6 h, 9 mg intra-arterial recombinant prourokinase plus low-dose heparin increased 90-day modified Rankin Scale 0–2 outcomes (40% vs. 25%; *P* = 0.04) and recanalization (66% vs. 18%; *P* < 0.001) compared with heparin alone. Intracranial hemorrhage with neurological deterioration occurred in 10% versus 2% (*P* = 0.06), while mortality was similar. The trial established proof of efficacy for primary pharmacological recanalization, but involved a selected pre-thrombectomy population and an early hemorrhagic signal (10).

POST-UK and POST-TNK addressed whether adjunctive intra-arterial lysis improves outcomes after near-complete or complete macrovascular reperfusion has been achieved by thrombectomy. Neither trial significantly improved 90-day disability-free survival (2, 3). By contrast, PEARL (intra-arterial alteplase) and ANGEL-TNK (intra-arterial tenecteplase) both reported higher 90-day mRS 0–1 rates after successful reperfusion (44.8% vs. 30.2% and 40.5% vs. 26.4%, respectively) (4, 5). The trials differed in reperfusion thresholds (eTICI 2c-3 vs. 2b-3), thrombolytic agent and dose, and cointerventions; neither PEARL nor ANGEL-TNK demonstrated a significant benefit across their other prespecified functional outcomes. These heterogeneous results do not negate PROACT II and do not establish routine post-thrombectomy adjunctive thrombolysis; primary lysis of an occluded artery must remain distinct from attempted treatment of residual distal or microvascular thrombi after reperfusion.

Native intra-arterial urokinase may still have an individualized rescue role for catheter- or device-related thrombus when arterial access is established, although randomized evidence is lacking. Overall, stroke applications are agent- and stage-specific: systemic native urokinase has receded, historical evidence supports primary intra-arterial recombinant prourokinase, and post-reperfusion adjunctive lysis has heterogeneous randomized evidence that is insufficient for routine use (2–5).

## 3 Intracranial compartments: local clot clearance

The strongest rationale for native urokinase is direct instillation into an intracranial blood collection. Once a catheter reaches the clot, local fibrinolysis and drain patency become more important than systemic fibrin selectivity. Although alteplase underpins much of the trial evidence, urokinase remains a lower-cost, readily available alternative in many neurosurgical units.

In intraventricular hemorrhage (IVH), urokinase delivered through an external ventricular drain may accelerate clearance and preserve catheter patency, but clinical evidence remains inconsistent. A retrospective study of 94 adults found no significant improvement in IVH severity, functional outcome, or shunt dependence with urokinase (11). In another retrospective cohort of 97 patients, urokinase and recombinant tissue-type plasminogen activator produced similar 180-day functional outcomes, although the latter achieved faster clearance and shorter dosing and drainage durations (12). Intraventricular urokinase should therefore be regarded as a pragmatic option rather than an established standard.

For spontaneous intracerebral hemorrhage (ICH), catheter aspiration followed by intracavitary urokinase directly targets residual hematoma. A 110-patient single-center series found the approach feasible, with reductions in hematoma volume and midline shift and few procedure- or lysis-related complications (13). In 55 patients with basal-ganglia hypertensive ICH, urokinase every 4 h rather than every 8 h shortened drainage and was associated with better 3-month outcomes (14). Both studies were nonrandomized; they support procedural feasibility and biological plausibility, not comparative efficacy or an optimal dosing regimen.

Chronic subdural hematoma (cSDH) may be the most intuitive niche because residual or septated blood and catheter obstruction can impair evacuation. In a retrospective cohort of 297 patients undergoing burr-hole drainage, postoperative subdural urokinase was associated with lower symptomatic recurrence (3.0% vs. 11.7%; *P* = 0.022), while infection and hemorrhagic complications were similar (6). A systematic review involving 1,449 patients reported low recurrence, infection, and intracranial bleeding rates with urokinase- or tissue plasminogen activator-facilitated drainage, but the evidence was heterogeneous and predominantly retrospective (15). Our case report of recurrent cSDH during dual antiplatelet therapy illustrates potential salvage use after reoperation, but cannot establish efficacy (7).

Across IVH, ICH, and cSDH, the procedural logic is consistent, but clinical signals are mixed and certainty is limited. Native urokinase may facilitate local clot liquefaction and catheter drainage at low cost; however, the available evidence is mainly retrospective, and it should not be presented as superior to alteplase or as a trial-proven standard.

## 4 Catheter-directed thrombolysis: selective vascular access

Catheter-directed thrombolysis (CDT) places a fibrinolytic within or adjacent to the thrombus. Native urokinase remains widely used in China and other resource-conscious settings because of cost, availability, and procedural familiarity, whereas many Western protocols use alteplase.

For lower-limb deep vein thrombosis (DVT), CDT is selective rather than routine. Randomized trials have not shown a major reduction in the overall incidence of post-thrombotic syndrome, although patients with acute iliofemoral DVT, severe symptoms, good functional status, and low bleeding risk may obtain earlier symptom relief and reduced syndrome severity (8). In a comparative study of acute iliofemoral DVT, AngioJet pharmacomechanical therapy plus adjunctive CDT required less urokinase and shortened catheter retention and hospitalization compared with CDT alone (16). However, a systematic review found substantial variation in agents, doses, infusion durations, adjunctive procedures, and outcome definitions (17). These data support individualized intervention, not routine urokinase-based CDT for proximal DVT.

The same principle applies to acute pulmonary embolism (PE). In intermediate-risk disease, CDT may improve thrombus burden and right-ventricular indices, but evidence for mortality or other hard outcomes remains uncertain. A retrospective comparison found similar short-term efficacy and safety with urokinase CDT and intraprocedural recombinant tissue-type plasminogen activator; the latter avoided prolonged catheter placement (18). During an alteplase shortage, a European single-center study also found comparable complication rates with urokinase and alteplase in ultrasound-accelerated treatment of intermediate–high-risk PE, suggesting that urokinase is a feasible substitute when alteplase is unavailable (19). Both studies were observational and do not establish therapeutic equivalence.

CDT therefore exemplifies the access component of urokinase's modern niche. Its rationale is direct, affordable delivery when intervention is justified—not pharmacological superiority over alteplase.

## 5 Intrapleural fibrinolysis: restoring drainage

In complicated parapneumonic effusion, empyema, and tuberculous pleurisy, fibrinous septations can prevent effective chest-tube drainage. Intrapleural urokinase aims to disrupt these loculations and restore drainage rather than produce systemic reperfusion.

A prospective randomized comparison found both urokinase and alteplase useful in complicated pleural effusion or empyema, with greater radiological efficacy for alteplase and a similar adverse-effect profile (9). A 2026 meta-analysis of 25 randomized trials involving 1,990 patients found that urokinase improved treatment success and drainage and shortened effusion absorption and hospitalization without significantly increasing adverse events; however, moderate study quality, dosing heterogeneity, and possible publication bias limited certainty (20). In tuberculous pleurisy, a 2024 meta-analysis likewise associated adjunctive urokinase with greater drainage, faster absorption, less residual pleural thickening, and improved pulmonary function, although substantial heterogeneity remained (21).

Intrapleural urokinase is therefore best presented as an affordable drainage adjunct to standard therapy, not as a replacement for antibiotics, chest-tube management, or surgery when escalation is required.

## 6 Catheter patency: prophylaxis and salvage

Thrombus, fibrin sheath, or intraluminal clot can obstruct hemodialysis catheters, central venous catheters, and implanted ports. After mechanical causes have been excluded, urokinase can be instilled into the lumen as a lock, dwell, or short infusion to target the obstruction with limited systemic exposure. Prophylaxis of recurrent dysfunction and salvage of an established occlusion should, however, be distinguished.

A review of hemodialysis access noted that taurolidine-based locks incorporating intermittent urokinase may reduce thrombotic dysfunction, but evidence remains limited (22). In a double-blind randomized trial of 68 high-risk hemodialysis patients, a once-weekly taurolidine–urokinase lock reduced dysfunction from 12.1 to 4.8 episodes per 1,000 catheter-days. It did not reduce overall lock-related costs and was associated with numerically more refractory thrombosis episodes (23). For salvage, the prospective multicenter PASSPORT 2 audit enrolled 117 hematology and oncology patients across eight centers. Patency was restored in 80% of interventions for partial withdrawal occlusion and 88% of interventions for total occlusion, with no recorded adverse events (24). Its uncontrolled audit design nevertheless limits causal efficacy inference.

Catheter-directed urokinase is therefore a practical local option for selected prophylaxis or access salvage, rather than evidence of systemic thrombolytic superiority.

## 7 Cerebral venous sinus thrombosis: a boundary case

Cerebral venous sinus thrombosis (CVST) tests the limits of the route-and-access framework because it is a venous thrombotic disorder rather than a drainage problem. Anticoagulation remains the foundation of treatment, with endovascular therapy reserved for carefully selected patients (25). In the 67-patient TO-ACT randomized trial, endovascular treatment plus standard care did not improve functional outcome over standard care alone, and the trial was stopped for futility (26). Evidence specifically supporting intrasinus urokinase is limited to uncontrolled combination therapies. A 10-patient series of hemorrhagic CVST reported recanalization without rebleeding or neurological deterioration after balloon-assisted thrombectomy, aspiration, and intrasinus urokinase, but the contribution of urokinase cannot be isolated (27).

Urokinase should therefore not be presented as standard CVST treatment. At most, it remains a potential rescue adjunct in selected patients who deteriorate despite anticoagulation, when endovascular access has been obtained and treatment is undertaken in an experienced center.

## 8 Recombinant prourokinase: renewed systemic reperfusion

Native urokinase and recombinant human prourokinase (rhPro-UK) should not be placed on a single therapeutic trajectory. rhPro-UK is the recombinant zymogen precursor of urokinase and is activated preferentially at the fibrin-rich thrombus surface, thereby limiting systemic plasminogen activation. It represents a pharmacological evolution that renews systemic reperfusion rather than extending the local-delivery niche of native urokinase.

In the open-label phase 3 PROST trial, intravenous rhPro-UK within 4.5 h of acute ischemic stroke onset was non-inferior to alteplase for 90-day excellent functional outcome (modified Rankin Scale score 0–1: 65.2% vs. 64.3%). Symptomatic intracranial hemorrhage rates were similar (1.5% vs. 1.8%), whereas overall systemic bleeding was lower with rhPro-UK (25.8% vs. 42.2%; *P* < 0.001); serious systemic bleeding was similar (28).

PROST-2, involving 1,552 patients, confirmed non-inferiority for excellent functional outcome (72.0% vs. 68.7%). It also found less symptomatic intracranial hemorrhage within 36 h (0.3% vs. 1.3%; *P* = 0.021) and less major bleeding within 7 days (0.5% vs. 2.1%; *P* = 0.0072) with rhPro-UK, while 7-day mortality was similar (29). A subsequent meta-analysis likewise found intravenous rhPro-UK broadly comparable to established thrombolytic comparators (30).

These findings challenge any generalized claim that the urokinase pathway has moved exclusively away from systemic reperfusion. However, both trials were open-label and conducted in China among patients without planned thrombectomy; they compared rhPro-UK with alteplase, not tenecteplase, and availability outside China remains limited. In addition, PUMICE found no superiority over standard care in mild ischemic stroke (31). The balanced interpretation is that native urokinase increasingly occupies local and access-driven niches, whereas recombinant prourokinase has renewed the pathway's systemic reperfusion potential without establishing universal replacement of alteplase or tenecteplase.

## 9 Discussion and future directions

The evidence supports a distinction between native urokinase and the broader urokinase pathway. For native urokinase, route, compartment, and access help explain its persistence: direct delivery can facilitate clot liquefaction, drainage, or catheter patency while limiting systemic exposure. Recombinant human prourokinase represents a parallel pharmacological evolution that has renewed systemic reperfusion potential and is supported by phase 3 stroke trials (28, 29).

This framework should not be mistaken for proof that local delivery is inherently effective. POST-UK and POST-TNK were neutral after successful endovascular reperfusion (2, 3), whereas PEARL and ANGEL-TNK reported higher rates of 90-day mRS 0–1 (4, 5). Differences in reperfusion thresholds, agents and doses, and cointerventions preclude a uniform conclusion; routine post-thrombectomy lysis therefore remains unestablished. TO-ACT did not demonstrate benefit from endovascular treatment in severe cerebral venous thrombosis (26). Most other applications reviewed here rely on retrospective cohorts, small single-center studies, or heterogeneous protocols. Biological plausibility, affordability, and familiarity therefore remain insufficient substitutes for comparative evidence.

Future studies should prioritize the settings with the strongest procedural rationale, including minimally invasive intracerebral hemorrhage evacuation, chronic subdural hematoma drainage, intrapleural fibrinolysis, and selected catheter-directed vascular therapy. Trials should standardize patient selection, dose, dwell time, catheter position, imaging and clinical endpoints, stopping rules, and bleeding definitions. Where feasible, native urokinase should be compared with alteplase or the standard care appropriate to each indication. For recombinant prourokinase, priorities include external validation beyond China, evaluation in thrombectomy-eligible populations, and direct comparison with contemporary agents such as tenecteplase. Health-economic and implementation analyses are also needed to determine when lower drug cost translates into meaningful system-level value.

## 10 Practical summary of clinical niches and reported regimens

To bridge the detailed discussion and the final conclusion, the following figure and table provide a concise synthesis of the review. Figure 1 presents the overarching conceptual message that urokinase remains clinically relevant mainly in local, catheter-directed, and compartment-defined settings, whereas Table 1 summarizes the reported regimens across major indications together with their therapeutic aims and supporting references.

**Figure 1:**
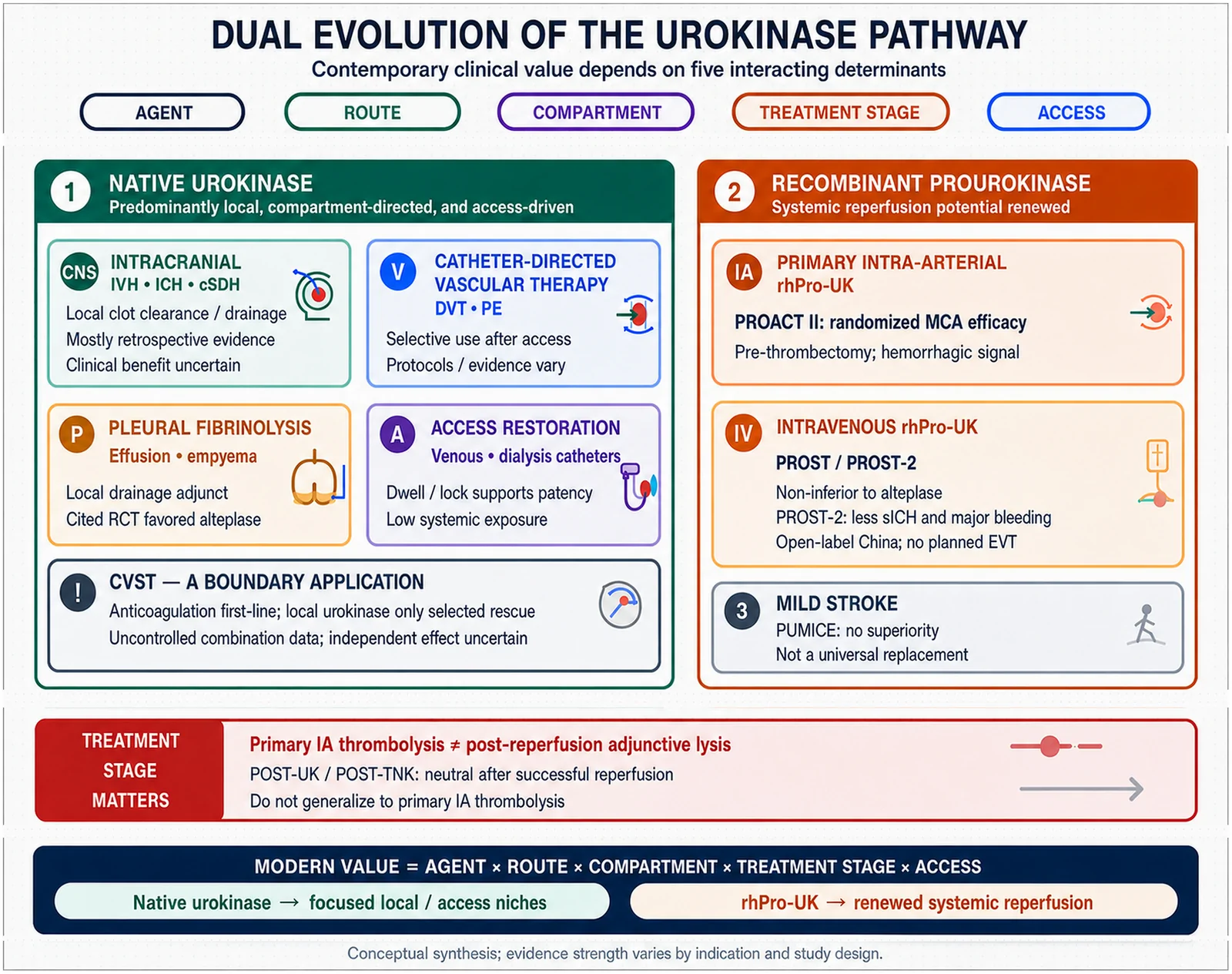
Dual evolution of the urokinase therapeutic pathway. Native urokinase now predominantly occupies local, compartment-directed, catheter-directed, and access-restoring niches, including intracranial clot evacuation, selected catheter-directed treatment of deep vein thrombosis and pulmonary embolism, intrapleural fibrinolysis, and catheter patency rescue. Recombinant human prourokinase follows a distinct reperfusion pathway: PROACT II provided historical randomized evidence for primary intra-arterial pharmacological recanalization, whereas PROST and PROST-2 demonstrated intravenous efficacy non-inferior to alteplase, with lower rates of symptomatic intracranial hemorrhage and major bleeding in PROST-2. For adjunctive post-thrombectomy thrombolysis, the evidence is heterogeneous: POST-UK and POST-TNK were neutral after eTICI 2c-3 reperfusion, whereas PEARL and ANGEL-TNK reported higher 90-day mRS 0–1 rates in broader eTICI 2b-3 populations (2–5). Agent identity, route, anatomical compartment, treatment stage, and access therefore jointly determine contemporary clinical value. Evidence for many local applications remains observational and heterogeneous. cSDH, chronic subdural hematoma; CVST, cerebral venous sinus thrombosis; DVT, deep vein thrombosis; ICH, intracerebral hemorrhage; IVH, intraventricular hemorrhage; PE, pulmonary embolism; rhPro-UK, recombinant human prourokinase.

**Table 1:** Reported regimens and evidence boundaries for native urokinase and recombinant prourokinase.

Clinical setting	Route or target	Reported regimen	Clinical role and evidence boundary	References
Native urokinase in acute ischemic stroke	Intravenous; systemic	1,000,000 IU (lower dose) or 1,200,000–1,500,000 IU (higher dose), within 6 h.	A pragmatic fallback when alteplase or tenecteplase is inaccessible. Evidence is retrospective; higher doses were associated with more extracranial bleeding.	(1)
Primary intra-arterial recombinant prourokinase for MCA occlusion	Intra-arterial; occluded MCA	9 mg over 2 h plus low-dose heparin, within 6 h (PROACT II).	Historical randomized evidence of efficacy before mechanical thrombectomy became standard; early hemorrhagic deterioration was more frequent. This strategy is distinct from post-reperfusion adjunctive lysis.	(10)
Adjunctive native urokinase after thrombectomy	Intra-arterial; reperfused target territory	Single 100,000-IU dose after near-complete or complete endovascular reperfusion (POST-UK).	Did not improve 90-day survival without disability; routine use of this specific regimen is not established.	(2)
Recombinant human prourokinase in acute ischemic stroke	Intravenous; systemic	35 mg within 4.5 h: 15-mg bolus over 3 min followed by 20 mg over 30 min.	PROST and PROST-2 showed non-inferiority to alteplase; PROST-2 reported less symptomatic intracranial hemorrhage and major bleeding. Trials were open-label and conducted in China among patients not proceeding to thrombectomy; PUMICE was neutral in mild stroke.	(28–31)
Intraventricular hemorrhage	Intraventricular; external ventricular drain	Study-specific repeated low-dose instillation; no standardized optimal dose or timing.	May facilitate clot clearance and drain patency, but evidence is mixed and benefits for functional outcome or shunt dependence remain uncertain.	(11, 12)
Spontaneous intracerebral hemorrhage	Intracavitary; hematoma catheter	5,000 IU every 6 h for up to 4 days in one series; 100,000 IU every 4 h versus every 8 h in a 55-patient study.	Feasible for local clot liquefaction after minimally invasive evacuation, but available studies are nonrandomized and do not establish an optimal schedule.	(13, 14)
Chronic subdural hematoma	Subdural; postoperative drain	Mean total dose 15,800 IU (range, 5,000–60,000 IU) over a mean of 2 days in one cohort; 20,000 IU in a salvage case.	Associated with lower recurrence in observational data and may restore drainage; evidence is heterogeneous and largely nonrandomized.	(6, 7, 15)
Acute iliofemoral deep vein thrombosis	Catheter-directed; thrombosed venous segment	Approximate total dose 5.82 million IU with CDT alone versus 1.80 million IU with AngioJet PCDT plus adjunctive CDT.	A selective option for acute, symptomatic iliofemoral disease in patients with low bleeding risk; agents, doses, devices, and endpoints remain heterogeneous.	(8, 16, 17)
Intermediate or intermediate–high-risk pulmonary embolism	Catheter-directed or ultrasound-assisted; pulmonary arteries	400,000–500,000 IU/day for 2–3 days in one catheter study; 300,000 IU per catheter over 10 h with ultrasound-assisted thrombolysis in another.	Observational studies support short-term feasibility when alteplase is unavailable, but comparative effects on major clinical outcomes remain uncertain.	(18, 19)
Complicated or loculated pleural effusion and empyema	Intrapleural; chest tube	100,000 IU three times daily for 2 days in one randomized trial; regimens varied across the meta-analysis.	A drainage-facilitating adjunct. Alteplase showed greater radiologic efficacy in the cited trial; pooled evidence is limited by protocol heterogeneity and possible publication bias.	(9, 20)
Tuberculous pleurisy	Intrapleural; chest tube	Dose and schedule varied; no unified regimen.	An adjunct to antituberculosis treatment. Meta-analysis suggested improved drainage, effusion absorption, residual pleural thickness, and pulmonary function, but included studies were heterogeneous.	(21)
Tunneled hemodialysis catheter prophylaxis	Intraluminal; catheter lock	Taurolidine–urokinase lock containing 25,000 IU once weekly.	Reduced thrombotic dysfunction from 12.1 to 4.8 episodes per 1,000 catheter-days in a randomized trial, without reducing overall lock-related costs; refractory thrombosis was numerically more frequent.	(23)
Occluded central venous catheter salvage	Intraluminal; dwell or push-lock	5,000 or 10,000 IU; up to 25,000 IU in some complete occlusions.	An audit reported restoration of patency in 80% of interventions for partial withdrawal occlusion and 88% for total occlusion, with no recorded adverse events. Mechanical causes should be excluded; controlled evidence is lacking.	(24)
Cerebral venous sinus thrombosis	Intrasinus; endovascular rescue	Individualized urokinase as part of balloon thrombectomy and aspiration; no standardized regimen.	Anticoagulation remains first-line. A possible rescue use is supported only by a 10-patient combination-therapy series, from which the independent effect of urokinase cannot be determined.	(25–27)

Regimens are reported dosing patterns from the cited studies, not prescriptive recommendations. Native urokinase and rhPro-UK are pharmacologically distinct. Dose selection must account for the indication, catheter position, clot burden, bleeding risk, institutional protocol, and local regulatory approval.

CDT, catheter-directed thrombolysis; cSDH, chronic subdural hematoma; EVD, external ventricular drain; IU, international units; MCA, middle cerebral artery; PCDT, pharmacomechanical catheter-directed thrombolysis; rhPro-UK, recombinant human prourokinase.

## 11 Conclusion

Native urokinase and recombinant prourokinase should not be placed on a single declining therapeutic trajectory. Their contemporary roles are better understood according to agent identity, route, anatomical compartment, vascular access, and treatment stage. The routine systemic role of native urokinase has narrowed, particularly where fibrin-selective agents are readily available. Nevertheless, native urokinase retains a practical role when it can be delivered directly to retained clot or fibrin obstruction, including intracranial blood collections, chronic subdural hematoma, selected catheter-directed vascular interventions, pleural loculations, and occluded vascular-access devices. In these settings, the therapeutic objective is usually not systemic reperfusion, but clot liquefaction, restoration of drainage or catheter patency, or localized thrombus reduction. Its low cost, availability, and procedural familiarity remain relevant, particularly in resource-conscious health-care systems.

Recombinant prourokinase represents a distinct and partly divergent development. Historical randomized evidence from PROACT II supports the biological and clinical potential of primary intra-arterial thrombolysis for an occluded cerebral artery, whereas PROST and PROST-2 have renewed interest in systemic reperfusion by demonstrating intravenous efficacy non-inferior to alteplase. The lower rates of symptomatic intracranial hemorrhage and major bleeding reported in PROST-2 further suggest a potentially meaningful safety advantage, although wider external validation and direct comparison with tenecteplase are required. For adjunctive post-thrombectomy lysis, POST-UK and POST-TNK were neutral, whereas PEARL and ANGEL-TNK reported higher 90-day mRS 0–1 rates (2–5). Differences in trial populations, reperfusion thresholds, and regimens mean that this evidence is heterogeneous; it neither supports routine use nor negates primary intra-arterial thrombolysis or other treatment stages.

The evidence supporting many local applications of native urokinase remains heterogeneous and is frequently based on retrospective studies, small cohorts, or region-specific protocols. Future studies should therefore standardize patient selection, dose, dwell time, catheter position, imaging endpoints, and safety definitions, while comparing urokinase with alternative fibrinolytics and drainage alone. Overall, the modern urokinase pathway reflects a dual evolution: native urokinase retains a focused local and access-driven niche, whereas recombinant prourokinase has re-established the pathway's potential in systemic stroke reperfusion.
