# Correction: Urokinase redux: route, compartment, access, and pharmacological evolution from native urokinase to recombinant prourokinase

**DOI:** 10.3389/fcvm.2026.2017216

**Published:** 2026-10-22

**Authors:** Frontiers Production Office

**Affiliations:** Frontiers Media SA, Lausanne, Switzerland

**Keywords:** catheter-directed thrombolysis, chronic subdural hematoma, intracerebral hemorrhage, local fibrinolysis, urokinase

The affiliation Beijing Tiantan Hospital, China was erroneously given as Massachusetts General Hospital and Harvard Medical School, United States for the reviewer Bowei Zhang.

The original version of this article has been updated.

